# Juvenile Coffee Leaves Acclimated to Low Light Are Unable to Cope with a Moderate Light Increase

**DOI:** 10.3389/fpls.2017.01126

**Published:** 2017-07-14

**Authors:** Claudine Campa, Laurent Urban, Laurence Mondolot, Denis Fabre, Sandrine Roques, Yves Lizzi, Jawad Aarrouf, Sylvie Doulbeau, Jean-Christophe Breitler, Céline Letrez, Lucile Toniutti, Benoit Bertrand, Philippe La Fisca, Luc P. R. Bidel, Hervé Etienne

**Affiliations:** ^1^Institut de Recherche pour le Développement (IRD), Unité Mixte de Recherche-Interactions Plantes Microorganismes Environnement, IRD, CIRAD, Université de Montpellier Montpellier, France; ^2^Institut National de la Recherche Agronomique (INRA)-Centre d’Avignon, UR 1115 Plantes et Systèmes de Culture Horticoles Avignon, France; ^3^Laboratoire de Botanique, Phytochimie et Mycologie, Faculté de Pharmacie, Unité Mixte de Recherche 5175 Centre d’Ecologie Fonctionnelle et Evolutive, Centre National de la Recherche Scientifique (CNRS) Montpellier, France; ^4^CIRAD, Unité Mixte de Recherche-Amélioration Génétique et Adaptation des Plantes Méditérranéennes et Tropicales Montpellier, France; ^5^Institut de Recherche pour le Développement (IRD), Unité Mixte de Recherche-Diversité Adaptation et Développement des Plantes, IRD, Université de Montpellier Montpellier, France; ^6^CIRAD, Unité Mixte de Recherche-Interactions Plantes Microorganismes Environnement, IRD, CIRAD, Université de Montpellier Montpellier, France; ^7^Institut National de la Recherche Agronomique (INRA), Unité Mixte de Recherche-Amélioration Génétique et Adaptation des Plantes Méditerranéennes et Tropicales Montpellier, France

**Keywords:** antioxidant activity, Arabica, coffee culture, phenolics, photoprotection, plasticity

## Abstract

The understorey origin of coffee trees and the strong plasticity of *Coffea arabica* leaves in relation to contrasting light environments have been largely shown. The adaptability of coffee leaves to changes in light was tested under controlled conditions by increasing the illumination rate on *C. arabica* var. Naryelis seedlings acclimated to low light conditions and observing leaf responses at three different developmental stages (juvenile, growing and mature). Only mature leaves proved capable of adapting to new light conditions. In these leaves, different major mechanisms were found to contribute to maintaining a good photosynthetic level. With increased illumination, a high photosynthetic response was conserved thanks to fast nitrogen remobilization, as indicated by SPAD values and the photorespiration rate. Efficient photoprotection was accompanied by a great ability to export sucrose, which prevented excessive inhibition of the Calvin cycle by hexose accumulation. In contrast, in younger leaves, increased illumination caused photodamage, observable even after 9 days of treatment. One major finding was that young coffee leaves rely on the accumulation of chlorogenic acids, powerful antioxidant phenolic compounds, to deal with the accumulation of reactive oxygen species rather than on antioxidant enzymes. Due to a lack of efficient photoprotection, a poor ability to export sucrose and inadequate antioxidant protection, younger leaves seemed to be unable to cope with increased illumination. In these leaves, an absence of induced antioxidant enzyme activity was accompanied, in growing leaves, by an absence of antioxidant synthesis or, in juvenile leaves, inefficient synthesis of flavonoids because located in some epidermis cells. These observations showed that coffee leaves, at the beginning of their development, are not equipped to withstand quick switches to higher light levels. Our results confirm that coffee trees, even selected for full sunlight conditions, remain shade plants possessing leaves able to adapt to higher light levels only when mature.

## Introduction

Among the numerous (around 120) wild species, all native to tropical and intertropical forests, making up the *Coffea* genus, only two have been selected for coffee production: *Coffea arabica* and *C. canephora*. Since the beginning of cultivation in Yemen or in Ethiopia (Harar) in the 14th century, *C. arabica* has generally been grown in full sunlight, in order to improve coffee bean quality and increase yield. It was recently shown that *C. arabica* full-sun trees from the first domestication center in Yemen form a genetic group called Harar-Yemen, to which the variety studied here belongs and which differs from the native wild coffee species (Klein et al., 2016). This ability to acclimate to full-sun light environments reflects the considerable plasticity of coffee leaves, highlighted in numerous eco-physiology studies (Nunes et al., 1993; Fahl et al., 1994; Da Matta, 2004).

As light is the main source of life for plants, they have developed a multilevel network to optimize the capture of light energy needed for optimum photosynthesis, whatever the light intensity (Nguyen et al., 1998; Martins et al., 2014a). When light energy suddenly exceeds that required for photosynthesis, an energy imbalance may occur, leading to photoinhibition and, under extreme conditions, to irreversible damage of the core components of the protein complex PSII. A concomitant rapid decrease in the photosynthetic electron transport rate occurs, preventing further production of reactive oxygen species (ROS) and might be considered as a mechanism of short-term adaptation to increased light. Persistent changes in light necessitate acclimation and the induction of a multilevel strategy to cope with the excess irradiation, with a view to controlling light absorption and dealing with the overflow of captured light energy. The first objective may be fulfilled by morphological and anatomical modifications, consisting in leaf area variation, leaf inclination or, in young leaves, an increase in trichome number, surface waxes, and cuticle or cell wall epidermis thickening (Jansen, 2002; Robson et al., 2015). The second is based on the establishment of a series of physiological adaptations set to fight against photo-oxidative stress. An immediate response consists in establishing a photoprotective mechanism with xanthophylls, carotenoids that remove excess excitation energy, dissipating excess energy by non-photochemical reactions (thermal reaction or alternative pathways) and activating ROS scavengers (antioxidant enzymes and protectants such as tocopherols or phenolic antioxidants) (Grace and Logan, 2000). However, excessive electron flows may also be rerouted toward photorespiration and other alternative electron sinks, like the Mehler reaction or nitrate reduction. Cyclic electron flow, a pathway possibly linked to chlororespiration, may be activated too and, by balancing energy and electron flows, may prevent ROS generation (Munekage et al., 2004). Later, during the acclimation step, general cell metabolism is modified leading, in particular, to the production of compounds with antioxidant properties, such as hydroxycinnamic acids and flavonoids (Bidel et al., 2007, 2015), which reduces the quantity of light entering the leaf (Day, 1993).

Following the work of Waterman et al. (1984) on *Barteria fistulosa*, numerous studies on rain-forest plants were conducted and showed a strong relationship between light and phenolic metabolism. The phenolic pool, including hydroxycinnamic acids and their esters (HCEs) such as chlorogenic acid (5-CQA), is generally greater in young leaves and in full-sun rather than in shade leaves, whatever the plants: understorey shrubs, semi-shade or full-sun grasses and trees (Mole et al., 1988; Islam et al., 2003; Ingersoll et al., 2010). Besides being defense compounds against herbivores, phenolics also protect leaves from photodamage, acting as light screens and as antioxidants when leaves are under oxidative pressure (Close and McArthur, 2002).

In coffee leaves, light absorption seems to be mainly controlled by morphological and anatomical plasticity (variation in leaf area and inclination) more than through physiological adaptations (Araujo et al., 2008). *C. arabica* leaves perform well at full exposure, despite a low photosynthetic rate when compared to other tropical tree species (Da Matta, 2004; Martins et al., 2014b). They usually dissipate the excess excitation energy through thermal or non-photosynthetic mechanisms to recover quickly from photoinhibition (Ramalho et al., 2000). Surprisingly, as shown by Chaves et al. (2008) when comparing leaves of *C. arabica* cultivated in the field under 48 and 100% natural light, ROS detoxification did not seem to occur through the classic key antioxidant enzymes, as their activity level was identical under both sets of growing conditions.

Coffee trees naturally accumulate a large quantity of phenolics from the HCE group. These compounds which are usually called chlorogenic acids, are described as potent antioxidants and considered to be involved in plant defenses, as their tissue concentration evolves under biotic or abiotic stress (Maher et al., 1994; Grace et al., 1998; Tegelberg et al., 2004). Analyses of HCE content in the different parts of the two cultivated species highlighted an intense accumulation of these compounds, together with caffeine, a purine alkaloid, in the developing green parts of the plants, i.e., the bud, the apical part of the stem, and the young pericarp (Campa et al., 2012). The apex of *C. canephora* plantlets appeared to be extremely rich in these compounds and more particularly in HCEs, which reached a value of around 14% of dry mass (Mahesh et al., 2007). Another potent antioxidant, mangiferin, a glycosylated xanthone, largely accumulates in *C. arabica* leaves (Campa et al., 2012). Flavonoids are phenolics that play a major UV-screening role in leaves. They are abundant in coffee leaves exposed to full sunlight conditions but their screening role has yet to be described in these organs, except in a recent study in which, unfortunately, their concentration was not given (Martins et al., 2014a).

All existing studies concern mature leaves from trees subjected to long periods of contrasting light conditions. Few data exist on the short-term response of coffee leaves to irradiance modifications and on the specific response of young coffee leaves which, being located at the top of the canopy, lie at the forefront when light conditions change. Mature, field-grown leaves have already been subjected to light changes during their growth; it is therefore to be expected that they have equipped themselves with a full range of mechanisms of adaptation to light changes (Müller-Xing et al., 2014). To date, despite numerous studies on mature leaves of trees grown in the field or greenhouse, the adaptive mechanisms put in place by young shaded coffee leaves to cope with fluctuations in light intensity, and more especially the role of the major leaf antioxidants, phenolics, have not yet been clearly elucidated.

The objective of the study presented here is to fill that gap. We chose to work under growth chamber conditions. Although the quality and intensity of light are different from natural conditions, growth chambers have the advantage of providing better control of environmental conditions. *C. arabica* plants were maintained for 3 months under low light conditions suitable for shade plants (classically used for coffee culture under growth chamber conditions and equivalent to 300 μmol photons m^-2^s^-1^) and then subjected to light increase in order to study adaptive responses. The adaptive mechanisms were analyzed for 9 days on leaves at three different developmental stages by focusing on the responses of the photosynthetic machinery, and the carbon and phenolic metabolisms.

## Materials and Methods

### Plant Material and Light Stress Experiment

Seeds of *C. arabica* var. Naryelis were harvested at the La Cumplida research center (Matagalpa, Nicaragua), depulped, fermented and dried to a 30% moisture content. Germination and plantlet conversion were performed at CIRAD (Montpellier, France) in growth chambers (65–75% humidity, 27°C day/22°C night, 12h photoperiod). Light was provided by Gavita HortiStar 600 SE EU (Holland) lamps, which were arranged to provide a photosynthetically active radiation (PAR) of 300 μmol photons m^-2^s^-1^ to the top of the plantlets. Low light regimes, similar to those encountered under shade (Araujo et al., 2008) are commonly used for this understorey species to grow plantlets in growth chambers (Thioune et al., 2017; Alves et al., in press). Water was supplied each day and plantlets were cultivated for 3 months under these conditions. Measurements were taken on leaves from 6-month-old plantlets (around 35 cm tall).

The experiment consisted in exposing the plantlets to an increase in light irradiance at day 0 and studying their physiological response over 9 days, leaving the other culture conditions unchanged. Before the light increase experiment, measurements were taken on day 0 (D0) and were used as a reference. During the experiment from day 0 after the first harvest to day 9, the lamps were placed closer to the plants but not excessively so as to avoid any modification of other environmental parameters such as temperature or relative humidity at the plant apex, as confirmed by control measurement. The light intensity was then set at 500 μmol photons m^-2^s^-1^.

On six dates of the kinetic [D0, D1, D2, D4, D7, D9 corresponding to control (0) and 1, 2, 4, 7, 9 days after starting to apply the increased light flow, respectively], five of the 35 plants were randomly selected to measure leaf gas exchanges and chlorophyll fluorescence and evaluate leaf antioxidant enzyme activities and biochemical content.

Three leaf developmental stages were chosen: juvenile and growing leaves from the first and second node below the apex (F1 and F2), and mature leaves (F3) from the third node (Supplementary Figure S1). These leaves differed in their average area, with F1 and F2 corresponding to 67 and 90% of the mean area of F3 leaves (F1 = 33.36 ± 4.35; F2 = 38.48 ± 1.65; F3 = 42.69 ± 1.77 cm^2^).

Alongside leaf photosynthesis and chlorophyll fluorescence measurements, the three types of leaves were collected after 5 h of lighting (at 2 pm local time) for further biochemical analyses from three different trees of both sets. Leaves were immediately frozen in liquid nitrogen, and kept at -80°C until analysis. The frozen material was directly used for enzymatic analyses or freeze-dried for subsequent metabolite analysis.

### Leaf Gas Exchange Measurements

Leaf photosynthesis was measured with a portable gas exchange measurement system (GFS-3000, Walz, Germany). Measurements of the net assimilation rate (A_net_) and of stomatal conductance (g_s_) were taken *in situ* between 10 am and 1 pm. The photon flux density was fixed at 300 μmol m^-2^s^-1^ of Photosynthetic Active Radiation (PAR) before the experiment and at D0, and increased to 500 μmol m^-2^s^-1^, a threshold value beyond which the lamps applied excessive heat to the leaves. CO_2_ concentration was set at 400 ppm, leaf temperature at 28°C, and relative humidity in the cuvette at 65%, and a constant flow rate through the cuvette of 800 ml min^-1^ was imposed. The 8 cm^2^ exchange area of the Walz cuvette was fully covered by leaves, and the environmental conditions in the leaf chamber were set to achieve a leaf-to-air vapor pressure deficit between 1.2 and 1.4 kPa. At the end of the gas exchange measurements, the light source was turned off for 4 min and the dark respiration rate was measured.

Then A_max_, which is an estimate of photosynthetic capacity, was measured under saturating conditions, at a photon flux density of 1500 μmol m^-2^s^-1^ of PAR, under a CO_2_ concentration of 1800 ppm.

Leaf chlorophyll and nitrogen content was evaluated on each of the leaves used, by measuring the mean of five readings from a portable chlorophyll meter (SPAD-502, Minolta, Japan).

### Chlorophyll Fluorescence Analysis

Chlorophyll fluorescence was measured immediately after each gas exchange measurement using a fluorescence module (PAM-fluorometer 3055-FL, Walz) attached to the gas exchange measurement equipment. The steady-state fluorescence yield (Fs) was measured after registering the gas-exchange parameters. A saturating light pulse (8000 μmol m^-2^s^-1^ during 0.8 s) was applied to achieve the light-adapted maximum fluorescence (*F*_m_′). The actual PSII photochemical efficiency (Φ_PSII_) was determined as Φ_PSII_ = (*F*_m_′-*F*_s_)/*F*_m_′.

### Analysis of the Fluorescent Transient Using the JIP-Test

Chlorophyll *a* fluorescence transients were measured at 2 pm with a Handy PEA chlorophyll fluorometer (Hansatech Instruments, King’s Lynn, Norfolk, United Kingdom) on the three types of leaves from five different plants at D0, D1, D2, D4, D7, and D9. For D1 plants, additional measurements were taken after 30, 60, and 90 min of increased light to observe the short-time fluorescence response kinetics. Leaves were dark-adapted for 20 min with a lightweight plastic leaf clip prior to measuring. The transients were induced by 1 s illumination with a LED array of six light-emitting diodes providing a fully saturating light intensity of 3000 μmol m^-2^s^-1^ and homogeneous irradiation. The fluorescence intensity at 50 μs was considered as *F*_0_ (Strasser and Strasser, 1995). The fast fluorescence kinetics (from *F*_0_ to *F*_m_, where *F*_0_ and *F*_m_ were, respectively, the minimum and maximum measured chlorophyll fluorescence of PSII in the dark-adapted state) were recorded from 10 μs to 1 s. The maximum quantum yield of photosystem II (PSII), the ratio of variable fluorescence (*F*_v_) to maximum fluorescence (*F*_m_), (*F*_v_/*F*_m_), the performance index (PI), a plant vitality indicator (Strasser et al., 2000; Strauss et al., 2006) and their components (*F*_v_/*F*_0_, RC/ABS which represents the ratio of reaction centers to the absorbance, (1–*V*_J_)/*V*_J_) where *V*_j_ is the relative variable fluorescence at time *J* = 2 ms) were calculated automatically. We also calculated the dissipated energy flux per PSII reaction center (DI_0_/RC), an indicator of the importance of processes other than trapping.

### Antioxidant Enzyme Activity and Lipid Oxidation Measurement

The fresh juvenile and mature leaves (F1 and F3) were ground in liquid nitrogen using a ball mill. The powder was homogenized in 4 ml of 100 mM potassium phosphate buffer, pH 7.8, containing 1 mM EDTA-Na_2_, 5 mM DTT (DL-Dithiothreitol, Sigma), 15 mM β-mercaptoethanol and 10% (wt./vol.) polyclar AT (insoluble polyvinylpyrrolidone BDH) for all the tested enzymes. The homogenate was centrifuged at 15 000 × *g* for 15 min and the supernatant was stored at 4°C.

Ascorbate peroxidase (APX; EC 1.11.1.11) activity was measured according to Nakano and Asada (1981) by monitoring the decrease in A_285_ at 25°C. The reaction mixture (3 ml) contained 50 mM potassium phosphate buffer (pH 7.0), 0.16 mM ascorbate, 0.1 mM EDTA-Na2, 3 mM H_2_O_2_ and extract. The concentration of oxidized ascorbate was calculated by using an extinction coefficient of 2.8 mM^-1^ cm^-1^. One unit of APX was defined as 1mmol ml^-1^ ascorbate oxidized min^-1^.

Glutathione reductase (GR; EC 1.6.4.2) activity was determined from the rate of NADPH oxidation as measured by the change in absorbance at 340 nm at 25°C as per Foyer and Halliwell (1976). The reaction mixture (3.0 ml) contained 100 mM tris buffer (pH 7.8), 2 mM EDTA-Na_2_, 50 μM NADPH, 0.5 mM GSSG and extract. GR activity was calculated using an extinction coefficient of 6.2 mM^-1^cm^-1^.

Monodehydroascorbate reductase (MDAR; EC 1.6.5.4) activity was determined spectrophotometrically by measuring the reduction of absorbance at 340 nm according to the technique described by Begara-Morales et al. (2015).

The 1.0 ml assay mixtures contained 50 mM TRIS-HCl (pH 7.8), 0.2 mM NADH, 1 mM ascorbate, and the sample. The reaction was initiated by adding 0.2 U of ascorbate oxidase (EC 1.10.3.3 from *Cucurbita*; Sigma-Aldrich, St. Louis, MO, United States), and the decrease in *A*340 due to NADH oxidation was monitored. One milliunit of activity was defined as the amount of enzyme required to oxidize 1 nmol NADH min^-1^ at 25°C.

Catalase (CAT; EC 1.11.1.6) activity was assayed as per Cakmak and Marschner (1992) in a mixture (3.0 ml) containing 100 mM sodium phosphate buffer (pH 7.0), 6 mM H_2_O_2_ and extract. At 25°C, the decomposition of H_2_O_2_ was monitored at 240 nm. CAT activity was calculated using an extinction coefficient of 39.4 M^-1^ cm^-1^.

To evaluate malondialdehyde (MDA) content, about 200 mg of powder was mixed with 1 ml of trichloracetic acid (TCA 0.1%) and then centifuged at 4°C for 15 min at 15 000 × *g*. Some (0.5 ml) of the removed supernatant was supplemented with 1 ml of thiobarbituric acid (0.5% TBA prepared in 20% TCA) and incubated for 30 min in a water bath at 95°C before stopping the reaction by immediate cooling in crushed ice for 30 min. In order to avoid the appearance of bubbles in the tubes, each sample was transferred to a haemolysis tube and butanol was added to the same volume as the sample. The colored butanol phases were recovered and their absorbance measured at 532 and 600 nm. After Substracting the non-specific absorbance read at 600 nm, the malondialdehyde concentration was calculated using its extinction coefficient (155 mM^-1^ cm^-1^).

The water-soluble protein content of extracts was measured by Bradford’s method (Bradford, 1976) using bovine serum albumin as a standard.

### Sugar Metabolism

Sugars were extracted from 20 mg samples of freeze-dried powder and measured by high-performance anion exchange chromatography coupled with pulsed amperometric detection (Dionex Chromatography Co.) as described by Dussert et al. (2006).

Starch content was determined on 30 mg of dry powder using the total starch kit GOPOD (D-glucose, K-Gluc, Megazyme International, Ireland). After elimination of soluble sugars and the soluble products of starch degradation, the residue was then hydrolysed into glucose units with α-amylase and amyloglucosidase successively. The D-glucose obtained was then degraded by a glucose oxidase and the hydrogen peroxide produced was quantified by spectrophotometry at 510 nm after a last enzymatic reaction. The results were expressed as a percentage of dry mass (% DW).

Analyses were carried out in triplicate by performing extractions on three independent plants.

### Phenolic Extraction and Quantification

The freeze-dried plant material was ground in a ball mill (TissueLyser II, Qiagen) and extraction was carried out by stirring 25 mg of plant material in 6 mL of MeOH/H_2_O (80:20, v/v) for 3 h at 4°C (225 rpm, Rotamax 120, Heidolph). After centrifugation (8 min, 3500 rpm), the methanol extract was collected and filtered (Millipore, 0.25 μm porosity) before analysis. Each extraction was carried out in triplicate. Each sample was characterized by its mean content of purine alkaloid (caffeine), HCE (CQAs, DiCQAs, FQAs), xanthone (mangiferin) and flavonoids ((+)-catechin, (-)-epicatechin, kaempferol, quercetin, and rutin), expressed as a percentage of dry weight. Quantification was carried out on 10 μL of extract using a HPLC system (Shimadzu LC 20, Japan) equipped with a photodiode array detector and consisting of an Eclipse XDB C18 (3.5 μm) column (100 mm × 4.6 mm, Agilent). The elution system (0.6 mL min^-1^) involved two filtered (0.2 μm pore size filter), sonicated and degassed solvents, namely solvents A (water/acetic acid, 98:2, v/v) and B (H_2_O/MeOH/acetic acid, 5:90:5 v/v/v). The linear gradient was: 0 min, 15% solvent B; 0–4 min, 25%; 4–8 min, 32%; 8–10 min, 35%; 10–14 min, 58%; 14–16 min, 62%; 16–18 min, 64%; 18–21 min 80%; 21–24 min 15%; 24–26 min, isocratic.

The calibration curve was plotted using three replicate points of standard solutions of caffeine, mangiferin, 5-CQA, from Sigma-Aldrich Chimie (St Quentin Fallavier, France), glucosylated kaempferol and quercetin, rutin, (+)-catechin, (-)-epicatechin and epigallocatechin from Extrasynthese (Lyon, France) and 3,5-diCQA from Biopurify Phytochemicals (Chengdu, China) at 25, 50, 75, and 100 μg mL^-1^. Identification was carried out by comparing spectra and retention times at 280, 320, and 360 nm (Supplementary Figure S4). Quantification of 3-, 4- and 5-CQA, FQAs and 3,4-, 3,5- and 4,5-diCQA was undertaken at 320 nm, caffeine and catechin derivatives at 280 nm, and mangiferin, kaempferol and quercetin derivatives at 360 nm, by comparison with respective standards. The presence of compounds was confirmed by analyzing one sample of each type of leaf by LC-MS, according to the technique previously described (Campa et al., 2012).

### Histochemical Analysis of Phenolic Compounds

Small pieces of freshly collected *Coffea* leaves were embedded in 3% agarose (type II EEO, Panreac) before cutting for histochemical examination. TS (40 μm) were obtained using a Leica VT 1000S vibrating blade microtome (frequency 7, speed 2). For mangiferin histolocalisation, TS were mounted in distilled water without any reagent. TS of specimens were viewed under a light microscope (Nikon Optiphot) with UV light (filter UV-1A: 365 nm excitation filter). Under these conditions, mangiferin exhibited strong yellow autofluorescence. Manual detachments of plant epidermis were carried out on abaxial epidermis to observe stomata before and after lighting to detect HCE. TS or epidermis detachments were directly mounted in DPBA reagent (2-aminoethyl diphenylborinate, Fluka). Under these conditions, HCEs were identified by greenish-white fluorescence and flavonoids (F) by yellow fluorescence, which could be reinforced using a specific filter B2a: 540 nm excitation filter (Mondolot-Cosson et al., 1997). Photographs were taken with a digital Nikon Coolpix 4500 camera. A microspectrofluorometer (Jobin-Yvon) equipped with an Olympus BX 60 microscope was used to obtain emission fluorescence spectra from fresh leaf TS previously immersed in DPBA reagent. An area of 5 μm in diameter was selected in the orange area of epidermal cells. Each leaf was analyzed in triplicate. Using a xenon lamp and monochromators, 361.5–368.5 nm wavelengths were produced to excite compounds. The subsequent fluorescence was detected with a charge couple device (CCD) camera (Hyper HAD-Sony CCD 300) and the fluorescence emission spectra were produced by the SpectraMax software package. A standard of rutin (Acros Organics, United States) was tested at 0.2% (w/v) concentration in DPBA reagent.

### Statistical Treatments

The results for leaf functional traits (maximum quantum efficiency of PSII) and metabolite content used to characterize leaf response to light increase were expressed as means (*n* = 5 biological replicates for chlorophyll fluorescence, *n* = 3 biological replicates extracted 3 times for compound analyses) ± standard deviation (sd). The results for leaf functional traits were analyzed using the Statistica software package (7.1 version, United States) to examine response variation, which was tested using a one-way analysis of variance (ANOVA). Means were compared between harvesting dates for each foliage stage and between leaf stages for the same harvesting date. When the *F*-test was significant, a Newman–Keuls test was carried out to compare means.

## Results

### The High Phenolic Content of Juvenile Leaves (F1) Decreases with Aging

The biochemical analysis confirmed that, whatever the developmental stage of the leaf, phenolics in the form of HCEs were particularly accumulated (**Table 1**). They were especially present in the form of 5-CQA (5-CQA) and 3,5-diCQA, at a concentration of 230 and 83 nmol mg^-1^ DW or 8.15 and 4.30% (mg/100 mg DW), respectively, in juvenile leaves (F1). Among the phenolics, mangiferin, a glucosylxanthone, and (-)-epicatechin, a flavonoid, also accumulated but less so (0.06 and 0.05 μmol mg^-1^ DW, respectively). Unlike what has been observed in leaves of other plant species, flavonoids were poorly represented, quantitatively and qualitatively, in the coffee leaves studied here. Only two flavanols, (+)-catechin and (-)-epicatechin, and one flavonol, rutin (quercetin-3-*O*-rutinoside), were present up to levels detectable by HPLC. The purine alkaloid caffeine appeared as the second most accumulated compound, with a content of 130 nmol mg^-1^ DW in juvenile leaves. As already observed, phenolics and alkaloids were more concentrated in young leaves (F1) than in growing and mature ones (F2 and F3, respectively), except for two minor HCEs, 4-CQA and one FQA isomer, and one flavonoid, (+)-catechin (**Table 1**).

**Table 1:** Quantitative data for alkaloids (caffeine) and phenolics in *Coffea arabica* var. Naryelis leaves at different growth stages.

	Compounds (μmol mg^-1^ dry weight)												
		Phenolics											
	Alkaloids	HCE							Xanthones	Flavonoids			
Leaf stage	Caffeine	5CQA	4CQA	FQA	3,4 Di	3,5 Di	4,5 Di	Total	Mangif.	Catechin	Epicat	Rutin	Total
1: Juvenile	130.0 a	230.2a	3.8a	0.9a	1.8a	83.3a	4.4a	324.4a	63.5a	15.9a	51.6a	0.3a	67.8a
2: Growing	91.7 b	148.1b	5.9a	1.7a	2.1a	16.1b	1.9ab	175.8b	21.0b	11.8a	37.1bc	0.1a	49.0b
3: Mature	73.6 c	83.9c	6.6a	2.5a	1.4a	4.2b	0.9bc	99.5c	17.1b	18.7a	25.3c	0.2a	44.2b

*Values are expressed as μmole mg^-*1*^ dry weight (DW) and correspond to the mean of three measurements from leaves of three different seedlings. Means followed by the same letter were not significantly different between leaf stage at *p* ≤ 0.05 (Newman–Keuls test). HCE, hydroxycinnamic acid esters, Mangif., mangiférine, Epicat, epicatechin. Total HCE represents the sum of 5- and 4-CQA, FQA and 3,4-, 3,5- and 4,5-diCQA and total flavonoids the sum of catechin derivatives and rutin.*

Histochemical observations of cross-sections in F1, F2 and F3 leaves, carried out before treatment, confirmed the rapid and marked decrease in mangiferin and HCE content during leaf development, especially observable in parenchyma cells (**Figures 8A,C,E,G**). Differences between F2 and F3 leaves were undetectable by microscopy analysis. Consequently, F2 cross-sections are not presented here. HCEs, which accumulated in every cell type of juvenile leaf tissue (F1, **Figure 8E**), appeared to be specifically localized in the adaxial, abaxial epidermis and in the palisade parenchyma of growing F2 and mature F3 leaves (**Figure 8G**). Flavonoids were not observed whatever the leaf growth stage (**Figures 8E,G**).

### Switching to More Intense Illumination has a Negative Impact on Photosynthesis for All Leaf Developmental Stages

**FIGURE 1:**
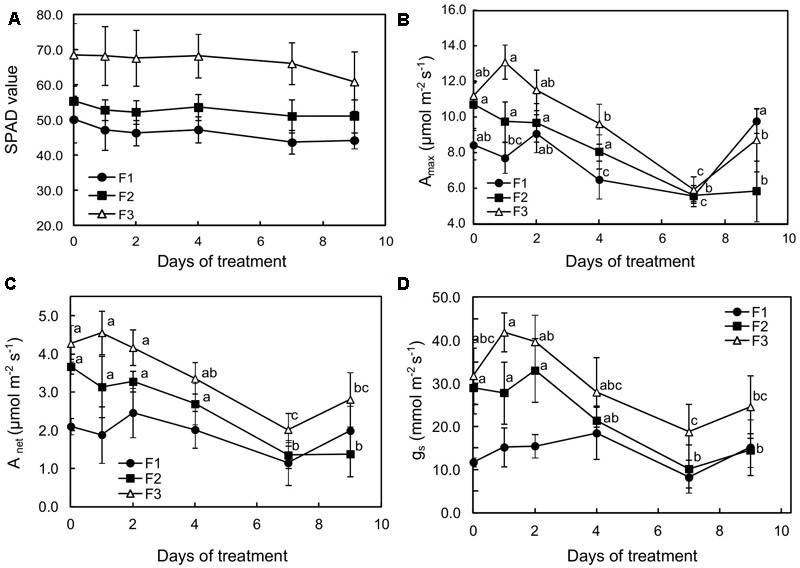
Effects of illumination increase (PAR 300 to 500) on **(A)** SPAD values, **(B)** photosynthetic capacity (A_max_), **(C)** net carbon dioxide assimilation, and **(D)** stomatal conductance (gs) of juvenile (F1), growing (F2), and mature (F3) *Coffea arabica* leaves. Error bars represent ± standard deviation of mean (*n* = 5). Different letters indicate significant differences at *p* ≤ 0.05 (Newman–Keuls test) among means for each leaf growing stage (absence of significant differences for SPAD values).

The three leaf stages also significantly differed in their nitrogen and chlorophyll content, as indicated by 1.9- and 1.37-fold higher SPAD values, estimated on a per unit area basis, in mature (F3) leaves than in growing (F2) and juvenile (F1) ones, respectively (**Figure 1A**). Histochemical observations showed that chlorophyll distribution varied too: it was present in most cells of the parenchyma of growing and mature leaves (F2 and F3), and was more specifically observable in palisade parenchyma of juvenile (F1) leaves (**Figures 8A,B**).

When plants were subjected to light increase, chlorophyll content (estimated by SPAD values) tended to slightly decrease in each leaf type, especially over the first 2 days of increased light in F1 and F2 leaves, although the values did not show statistical differences for the same leaf development stage (**Figure 1A**). For F2 and F3 leaves, photosynthetic capacity (maximum assimilation at saturating light and CO_2_: A_max_), net carbon dioxide assimilation (A_net_) and stomatal conductance (g_s_) drastically decreased over the first 7 days of higher illumination, and then increased on day 9 (**Figures 1B–D**). This effect was particularly noticeable in mature leaves (F3) in which all these variables were significantly lower (about twofold) after 7 days’ exposure to higher illumination, reaching 5.92 and 2.02 μmol m^-2^s^-1^ CO_2_ fixed, respectively for A_max_ and A_net_, and 20 mmol H_2_O m^-2^s^-1^ for g_s_. It should be noted that, according to the A_max_ and A_net_ values, photosynthesis was slightly stimulated in F3 and F1 leaves on day 1 and 2, respectively, after light was increased. In the youngest leaves (F1), despite a non-significant 20% decrease in photosynthetic capacity after 7 days of light increase, A_net_ and g_s_ increased up to day 4 (**Figures 1C,D**). A_net_ and g_s_ were substantially lower in F1 leaves when compared to the other leaves at the beginning of the observation period.

### Switching to More Intense Illumination Results in Photodamage in Juvenile and Growing Leaves

**FIGURE 2:**
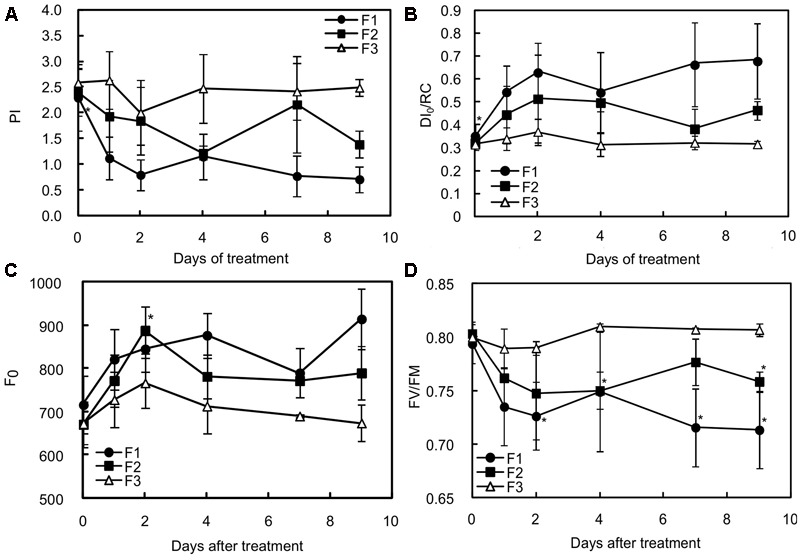
Effects of illumination increase on different fluorescence parameters in juvenile (F1), growing (F2) and mature (F3) *C. arabica* leaves. **(A)** Represents the values of the performance index (PI), **(B)** the maximum quantum rate (*F*_V_/*F*_M_), **(C)** the non-photochemical energy dissipation per reaction center (DI_0_/RC), and **(D)** the minimum fluorescence level after dark acclimation (*F*_0_). Error bars represent ± standard deviation of mean (*n* = 5). Significant differences at *p* ≤ 0.05 (Newman–Keuls test) among means for each leaf growing stage are noted only in juvenile (F1) and growing (F2) leaves and mentioned by an asterisk.

Unlike mature (F3) leaves, juvenile and growing (F1 and F2) leaves clearly appeared to have been stressed, as indicated by the decrease in the PI and in *F*_v_/*F*_M_ (**Figures 2A,D**). According to Adams et al. (2006), the small *F*_v_/*F*_m_ decrease observed in mature leaves can be considered as photoprotection while the more substantial decreases in juvenile and growing leaves may indicate photodamage. PI is a multi-parametric expression of three independent steps contributing to photosynthesis, namely RC/ABS, *F*_v_/*F*_0_ and (1-*V*_J_)/*V*_J_. The reduction in PI was particularly found in the youngest leaves (F1) for which the values of the three independent variables were statistically different before (at day 0) and 7 days after increasing illumination (**Table 2**). In F1 and F2 leaves, the lower PI values were mainly attributable to a decrease in RC/ABS and *F*_v_/*F*_0_. The decrease in RC/ABS reflected the down-regulation of PSII reaction centers, a well-known mechanism of leaf adaptation to light (Lu et al., 2001). The decrease in *F*_V_/*F*_0_, amounting to 33.59 and 23.13%, respectively, in F1 and F2 leaves, exposed to higher light for 9 days, suggested a reduction in energy trapping probability, in the sense of the quantum yield of primary PSII photochemistry. The severe decrease in (1-*V*_J_)/*V*_J_ values observed in F1 leaves (and not preceded by a slight increase on the 1st day, as observed in F2 and F3 leaves) may be interpreted as the consequence of a reduced capacity to convert excitation energy into photosynthetic electron transport. On the other hand, the increase in DI_0_/RC observed in F1 and F2 treated leaves showed that the rate of energy flux dissipation by processes other than trapping, such as heat dissipation, increased in these leaves (**Figure 2B**).

**Table 2:** Effects of increased irradiance on the evolution of three fluorescence parameters contributing to the performance index value (PI) in *C. arabica* leaves at three different growing stages.

	Juvenile leaf (F1)			Growing leaf (F2)			Mature leaf (F3)		
Time (days after treatment)	RC/ABS	*F*_v_/*F*_0_	(1-*V*_j_)/*V*_j_	RC/ABS	*F*_v_/*F*_0_	(1-*V*_j_)/*V*_j_	RC/ABS	*F*_v_/*F*_0_	(1-*V*_j_)/*V*_j_
0	0.593a	3.873a	1.183a	0.617a	4.086a	1.132ab	0.643a	4.005a	1.211a
1	0.496bc	2.842b	0.915ab	0.569a	3.251ab	1.207ab	0.628a	3.776a	1.324a
2	0.444c	2.689b	0.773b	0.514a	3.032ab	1.381ab	0.576a	3.754a	1.086a
4	0.476c	3.135ab	0.925ab	0.527a	3.016b	0.935 b	0.619a	4.259a	1.110a
7	0.442c	2.610b	0.761b	0.592a	3.514ab	1.208ab	0.602a	4.176a	1.145a
9	0.429c	2.572b	0.751b	0.523a	3.141ab	1.005ab	0.612a	4.181a	1.166a

*Data presented are means of 4 to 6 values. Different letters indicate significant differences between means from each type of leaf between sampling dates (*p* ≤ 0.05, Newman–Keuls test).*

This increase in DI_0_/RC suggests that F1 and F2 leaves were under greater pressure than F3 leaves exposed to higher light intensity. It does not tell us whether the dissipation of energy flux by processes other than trapping was effective in preventing overheating and damage to photosystems. The gradual increase in the initial value of the minimum fluorescence rate (*F*_0_) in F1 and F2 leaves from 24 h of higher light exposure (**Figure 2C**) therefore suggests that all the triggered adaptive mechanisms were not enough to prevent the photosynthetic machinery from being damaged.

### Switching to More Intense Illumination Drastically Affects Sugar Metabolism but Only Slightly Affects the Respiration Rate in Juvenile and Growing Leaves

**FIGURE 3:**
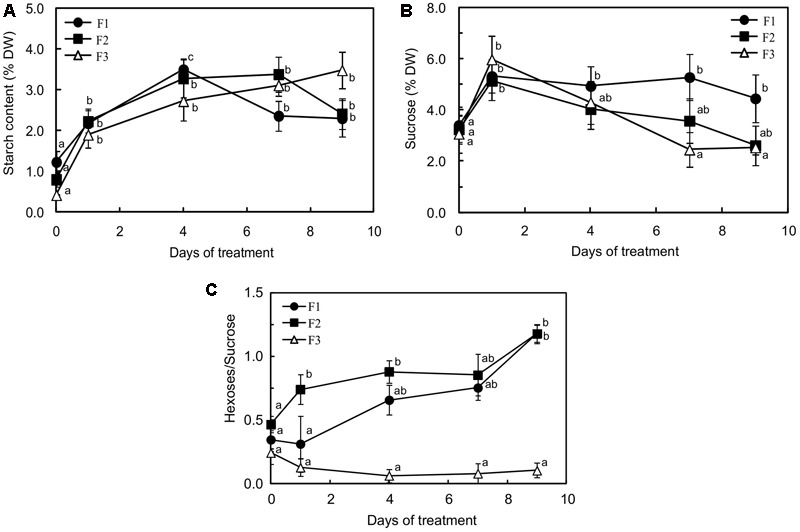
Starch **(A)**, sugar **(B)** content and changes in hexose/sucrose **(C)** in juvenile (F1), growing (F2) and mature (F3) *C. arabica* leaves subjected to an illumination increase (PAR 300 to 500) for 9 days. Error bars represent ± standard deviation of mean (*n* = 5). Different letters indicate significant differences at *p* ≤ 0.05 (Newman–Keuls test) among means for each leaf growing stage.

Starch accumulation was observed at all leaf developmental stages as a consequence of the exposure to more intense illumination (**Figure 3A**). However, there were differences. The concentration in starch was the lowest in F3 leaves before the light flux increase, suggesting that export was more active than at the youngest development stage. Over the first 3 days of increased light, higher starch accumulation was observed in F1 and F2 leaves (2.95- and 1.91-fold higher, respectively) reflecting a greater need to deal with low exports. Throughout more intense light exposure, the starch content regularly increased in F3 leaves, amounting to around 1.5-fold higher than that of F1 and F2 leaves after 9 days of the experiment, as starch accumulation ceased in those leaves after 4 and 7 days, respectively.

A drastic increase in sucrose content was observed in all the leaves 1 day after exposure to higher light (1.43-, 1.63-, and 1.95-fold higher in F1, F2 and F3 leaves, respectively) (**Figure 3B**). The sucrose content then decreased rapidly in F3 and F2 leaves, reaching the value before light change from day 7 and from day 9, respectively. In F1 leaves, the decrease was only observed after 7 days of treatment and, after 9 days, the sucrose content remained 1.4-fold higher than before treatment. In mature leaves, the increase in starch accumulation and the decrease in sucrose content caused by increased light were accompanied by a clear decrease in glucose content (sevenfold less on day 9 than before treatment) and a return to the previous concentration in fructose after a drastic increase 1 day after treatment (Supplementary Figures S2A,B). Glucose, which was weakly accumulated in mature leaves, showed the highest concentrations before light increase, and fructose 1 day after exposure, reaching values of 0.61 and 0.44% DW, respectively. Interestingly, F3 leaves displayed a higher fructose than glucose content, throughout the period of stress (Supplementary Figures S2A,B).

In juvenile (F1) and growing leaves (F2) leaves, fructose, and more especially glucose, contents markedly increased and amounted to less than 5% of the sucrose content (**Figure 3C**). In juvenile leaves, the glucose content (Supplementary Figure S2A) practically reached that of sucrose (4.06 and 4.45% DW, respectively). The hexose to sucrose ratio was higher in F1 and F2 leaves than in F3 leaves before exposure to higher light and the difference from mature F3 leaves greatly increased during the treatment (**Figure 3C**), reflecting durable and huge inhibition of sucrose biosynthesis, which reduced the photosynthetic capacity in the younger leaves.

**FIGURE 4:**
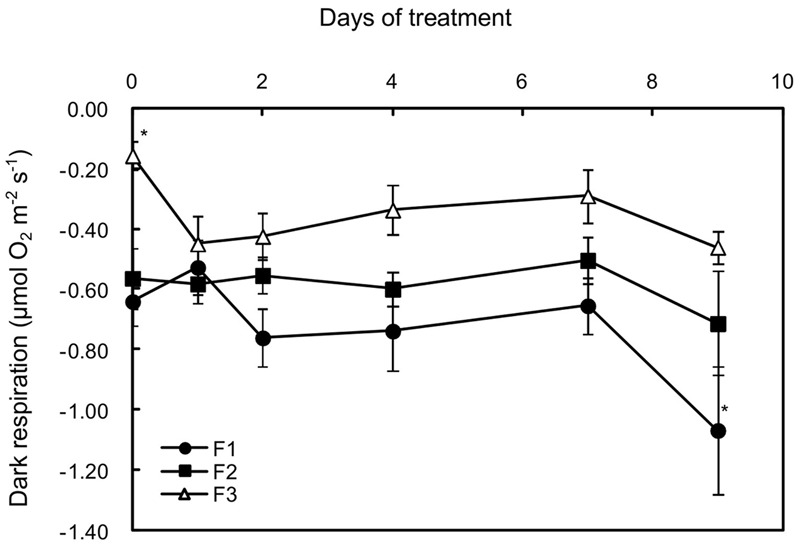
Effects of illumination increase on the dark respiration rate (R*d*) in juvenile (F1), growing (F2), and mature (F3) *C. arabica* leaves. Error bars represent ± standard deviation of mean (*n* = 5). Significant differences at *p* ≤ 0.05 (Newman–Keuls test) among means for each leaf growing stage are indicated by an asterisk.

As usually observed in leaves of evergreen or deciduous species (Villar et al., 1995), the dark respiration rate (R*d*) decreased with increasing leaf age, being threefold lower in mature leaves than in the other two types on D0 of treatment (**Figure 4**). Increasing light was followed by a twofold increase in R*d* for mature leaves (F3) and then the rate was maintained for 6 days. A moderate increase (not significant) was also observed in juvenile leaves (F1) from day 2 to day 7, while R*d* was not affected in growing leaves (F2) until day seven. However, on day nine, a large increase in dark respiration was observed in all of the leaves.

### Even in Mature Leaves, Switching to More Intense Illumination Slightly Modifies the Antioxidant Enzyme Activity

**FIGURE 5:**
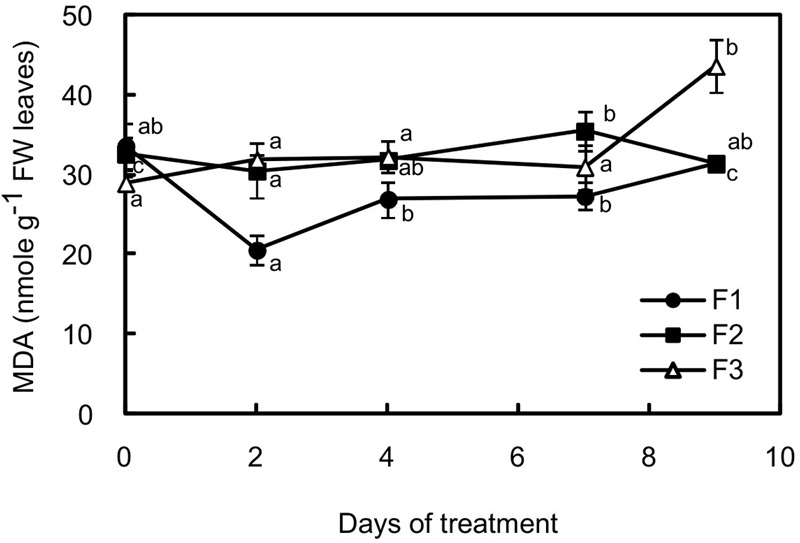
Changes in malondialdehyde (MDA) content in juvenile (F1), growing (F2) and mature (F3) leaves of *C. arabica* plantlets subjected to an illumination increase (PAR 300 to 500) for 9 days. Error bars represent ± standard deviation of mean (*n* = 5). Different letters indicate significant differences at *p* ≤ 0.05 (Newman–Keuls test) among means for each leaf growing stage.

As an indicator of oxidative stress, the malondialdehyde content was evaluated in the three types of leaves (**Figure 5**). Surprisingly, the content in reactive species derived from lipid peroxidation slightly increased in F3 leaves with more intense illumination, while it decreased in F1 leaves, reaching the lowest value after 2 days (1.63-fold lower than before stress).

**FIGURE 6:**
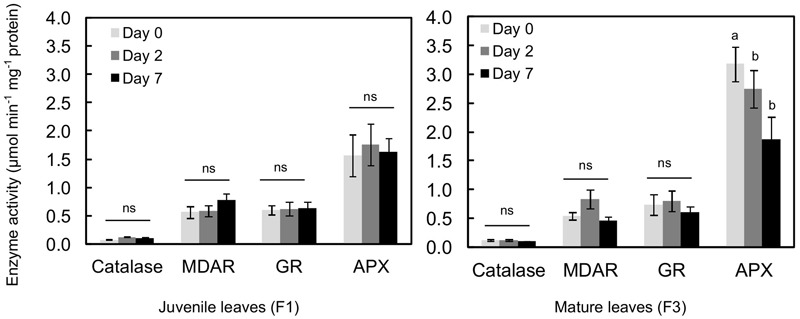
Antioxidant enzyme activity in juvenile (F1) and mature (F3) leaves of *C. arabica* plantlets before and after 2 and 7 days of increased illumination (PAR 300 to 500). Error bars represent ± standard deviation of mean (n = 3 for catalase, 4 for APX and 6 for MDAR and GR). Different letters indicate significant differences at p ≤ 0.05 (Newman–Keuls test) among means for each leaf growing stage. MDAR, monodehydroascorbate reductase; GR, glutathione reductase; APX, ascorbate peroxidase.

The activities of an antioxidant enzyme (catalase) and enzymes of the recycled oxidized forms of antioxidant such as glutathione and ascorbate [MDAR, APX, and GR] were evaluated in all the leaves, before and after treatment (**Figure 6**). Catalase, MDAR and GR activities were similar in juvenile (F1) and mature (F3) leaves before exposure to higher illumination. Only APX activity was twice as high in mature leaves as in juvenile ones. Exposing plantlets to more intense illumination had a very low impact on enzyme activity, especially in F1 leaves. In F3 leaves, except after day 2 where a slight increase in MDAR activity was observed, increasing light even resulted in a decrease in enzyme activity. This effect was particularly marked for APX, whose activity was 1.7-fold lower after 7 days of higher light.

### Switching to More Intense Illumination Induces a Rapid Increase in HCEs in Mature Leaves and Localized Synthesis of Flavonoids in Juvenile Leaves

**FIGURE 7:**
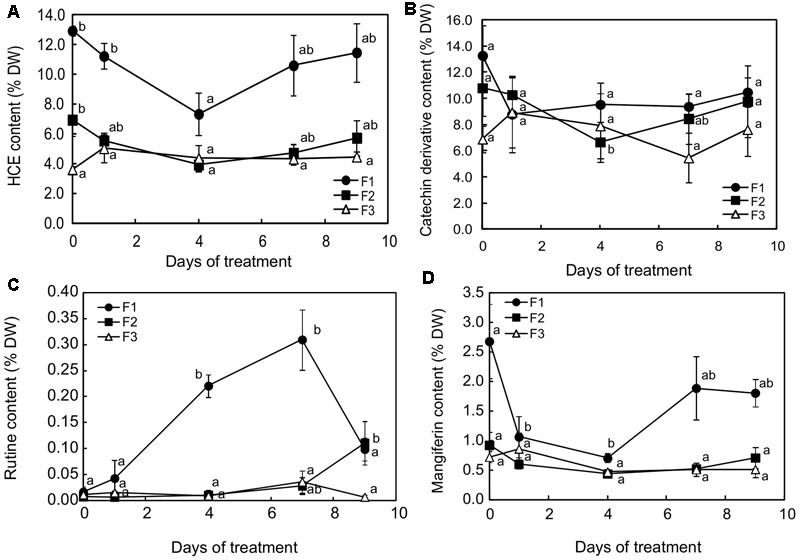
Contents for **(A)** hydroxycinnamic esters (HCEs), **(B)** catechin derivatives, **(C)** rutin and **(D)** mangiferin in juvenile (F1), growing (F2) and mature (F3) leaves of *C. arabica* plantlets subjected to increased illumination during 9 days. Error bars represent ± standard deviation of mean (*n* = 3) of two replicates. Different letters indicate significant differences at *p* ≤ 0.05 (Newman–Keuls test) among means for each leaf growing stage.

Changes in caffeine and phenolic content were monitored after 1, 4, 7, and 9 days of higher light at the three leaf stages. Whatever the leaf development stage, a modification in metabolite content was observed as early as 24h after light intensity increased (Supplementary Figure S3 and **Figure 7**). Caffeine, total catechin ((+)-catechin and (-)-epicatechin) and mangiferin content (Supplementary Figure S3 and **Figures 7B,D**, respectively) generally increased in F3 leaves and decreased in F1 and F2 leaves, so that all the leaf stages displayed almost similar contents for these compounds from day 1 to day 4. The phenomenon was particularly marked between F2 and F3 leaves, which showed equivalent contents for caffeine, total catechin, rutin, HCEs and mangiferin throughout the exposure period, except after 4 days for the total catechin content and after 7 days for rutin (**Figures 7B,C**). The most marked response to higher light was observed in juvenile (F1) leaves, in which the HCE and mangiferin contents strongly decreased up to day 4 (**Figures 7A,D**) and rutin was highly accumulated from day 1 to day 7 (**Figure 7C**). After 4 days of more light increase treatment, 3,5-diCQA, a DiCQA, and mangiferin contents were fourfold lower than initially (1.21 and 0.70% DW instead of 4.30 and 2.68% DW, respectively).

**FIGURE 8:**
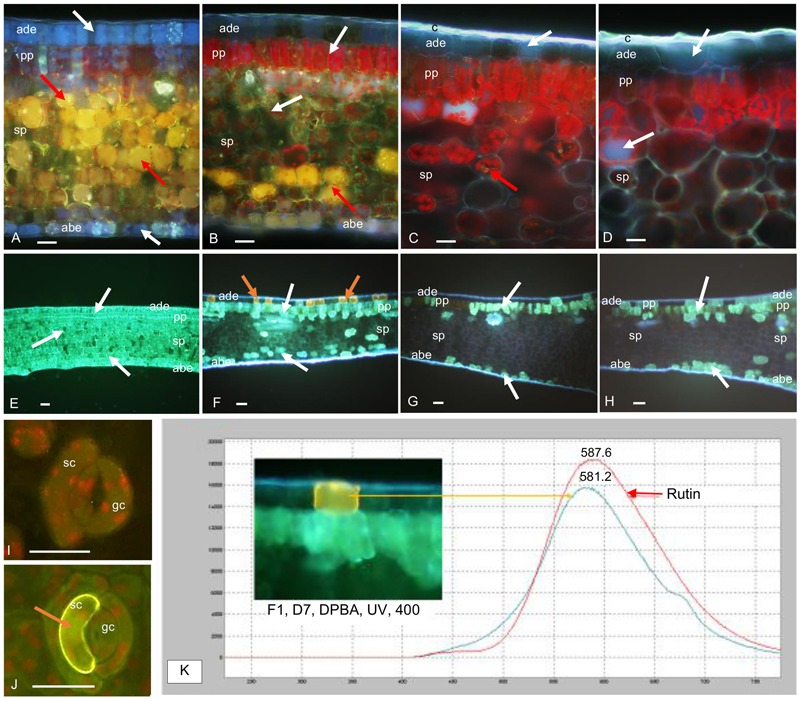
Transversal sections of juvenile (F1) and mature (F3) *C. arabica* leaves before (D0) and after 7 days (D7) of increased illumination. **(A–D)** Mangiferin (M) and HCE distribution in F1 and F3 leaves at D0 and D7, mounted in distilled water, observed under UV light (365 nm), x400: **(A)** in F1 leaves at D0, M (bright yellow natural fluorescence, red arrows) is specifically concentrated in the vacuoles of spongy parenchyma (sp) cells. HCEs, (blue fluorescence, white arrows) are concentrated in adaxial (ade) and abaxial epidermal (abe) cells and in palisade (pp) and spongy parenchyma in which their natural blue fluorescence is masked by the strong yellow fluorescence of M. Chlorophyll red fluorescence is also attenuated by M and HCE fluorescences. **(B)** In F1 leaves at D7, M is only detectable in the sp cells (red arrows). The presence of HCEs is slightly visible in ade and abe cells. In pp, their natural fluorescence is masked by chlorophyll fluorescence. **(C)** In F3 leaves at D0, M is almost undetectable and HCEs appeared less concentrated than in F1 leaves (white arrows); the cuticle (c) is thick compared to that of F1 leaves. **(D)** In F3 leaves at D7, M is almost undetectable and HCEs appear as concentrated as at D0 (white arrows). **(E–H)** HCE and Flavonoid (F) distribution in F1 and F3 leaves at D0 and D7, mounted in DPBA, observed under UV light (365 nm), ×100: **(E)** confirms the accumulation of HCEs (white arrows) in all the tissues of F1 leaves at D0. F are not detectable. **(F)** In F1 leaves at D7, HCEs are localized in some ade, abe and sp cells. They seem to be particularly accumulated in pp cells (white arrows). Some cells from ade present high flavonoid (F, orange arrows) concentration. **(G)** In F3 leaves at D0, HCEs only accumulate in very few ade cells, and in some pp and abe cells (white arrows), F are not detectable. **(H)** In F3 leaves at D7, HCEs seem distributed in more ade and abe cells compared to D0 (white arrows). **(I,J)** Flavonoid (F) distribution in abaxial epidermis and stomata cells of F1 leaves at D0 and D7, mounted in DPBA, observed under blue light (450–490 nm), x 400: **(I)** at D0, F are almost undetectable in guard cells (gc) and subsidiary cells (sc) of the stomata. **(J)** at D7, a strong yellow fluorescence indicates Flavonoid accumulation in a sc (orange arrow). **(K)** Fluorescence emission spectra of an adaxial epidermal cell concentrated in F1 at D7, and rutin standard, obtained under excitation at 365 nm, in DPBA: maxima emission peaks are near each other (581.2 nm for cell content and 587.6 nm for rutin).

Microscopic observation of cross-sections of F1 leaves exposed to higher light for 7 days showed that the distribution of mangiferin and HCEs evolved differentially in leaf tissues after higher illumination (**Figure 8**). When mangiferin seemed almost to vanish from spongy parenchyma cells (**Figure 8B**), the HCE level mostly decreased in spongy parenchyma, and in adaxial and abaxial epidermis cells (**Figure 8F**). Cells from abaxial and adaxial epidermis displayed yellow fluorescence under UV light in DPBA buffer, indicating flavonoid presence (**Figure 8F**). The fluorescence spectra emitted by these cells was analyzed by microspectrofluorometry and confirmed the presence of rutin (**Figure 8K**). Comparing abaxial epidermis under blue light in DPBA buffer before and after 7 days of higher light revealed that flavonoid-accumulating cells often corresponded to subsidiary cells of closed stomata (**Figures 8I,J**). In F3 leaves, except for the presence of HCEs in numerous epidermis cells, leaves stressed for 7 days did not show any marked differences compared to that before the light increase (**Figures 8D,H**).

## Discussion

### Mature Leaves Are Able to Cope with Increased Illumination, Arguably Because of Efficient Photosynthesis Associated with Effective Photoprotection

As already seen here, maintaining a high level of photosynthetic capacity for mature leaves (F3) over 7 days indicated that increasing light did not damage the photosynthetic machinery of those leaves. As highlighted by previous evaluations on *C. canephora* leaves (Netto et al., 2005), SPAD evaluations indicated that the photosynthetic pigment content was conserved and that nitrogen allocation, a key factor for the process of acclimation to intense light in coffee plants (Ramalho et al., 1997), was always effective. Moreover, the mature leaf contents for antioxidant compounds such as flavonoids and HCEs was not greatly modified, showing that higher light slightly triggered adaptive mechanisms, acting on the HCE and catechin isomer content. The HCE content increased after 24 h and was maintained at this level during application of the higher light regime. This increase in HCE content was exclusively due to a greater accumulation of two HCEs out of the six present in *C. arabica* leaves, 5-CQA and especially 3,5-DiCQA (data not shown). Their content was 1.3- and 2.6-fold higher after 1 day of increased light exposure, respectively. In mature leaves, fructose and sucrose contents were also the highest 24 h after exposure to light increase. Grace and Logan (2000) showed that feeding tomato plants (HCE accumulating plants) with sucrose led to an increase in chlorogenic acid (5-CQA) content in leaves, indicating that a high soluble sugar level stimulated carbon flow through the phenylpropanoid pathway, which leads to phenolic synthesis. The narrow relation between photosynthesis/carbon source and the phenylpropanoid pathway was demonstrated by a 20–50% decrease in HCE content in transformed tobacco plants underexpressing transkelatose (20 to 40% less), an enzyme from the Calvin cycle (Henkes et al., 2001).

Microscopic observations revealed the presence of HCEs exclusively in epidermis and palisade parenchyma cells of mature leaves rather than systematic accumulation in all cells of the leaf blade in juvenile leaves. This specific distribution of antioxidant compounds in nearby tissues of the leaf surface suggests that cellular differentiation occurred during leaf aging. This helps to maintain on the leaf surface high proportions of chlorogenic acid and derivatives, potential scavengers of free radicals and other oxidative species generated by environmental stresses (intense light, low temperatures or pathogen infection). Grace and Logan (2000) considered that chlorogenic acid, in addition to its role of an alternative carbon sink under excess light conditions, may actively participate, as a potent hydrogen-donating antioxidant, in photoprotection due to its ability to consume photochemical reduction power. In the event of light increase, its presence in the zone of active photosynthesis may help classic ROS (SOD, APX, MDAR or GR) in radical scavenging when their capacity is exceeded. By comparing *C. arabica* leaf metabolism between shaded and sunlit plants, Martins et al. (2014b) concluded that the priority for coffee plants is to adjust leaf metabolism to fight against oxidative stress rather than to benefit from extra light enhancement in photosynthetic yield.

Another important modification observed in leaf phenolics concerned catechin derivatives, whose content in mature leaves decreased 3-fold between the beginning and day 7 of higher light exposure. By feeding *Arabidopsis* plantlets with (+)-catechin at different concentrations, Rani et al. (2011) found an accumulation of this compound in leaves, accompanied by an increase in water potential, leaf thickness, and stimulation of overall growth. Reducing leaf growth by strongly decreasing (+)-catechin concentration could be the response of mature leaves to higher light intensity. This is in accordance with classic observations of full-sun coffee plants that have more small leaves than shaded plants (Voltan et al., 1992).

Microscopy also revealed that, under a constant light regime (D0), only growing and mature leaves possessed a thick cuticle wax. This protective layer of adaxial and abaxial surfaces seemed to be set up during stress in juvenile leaves. Cuticle may be an additional protection in older leaves, limiting the intensification of water evaporation due to increased radiation. Nevertheless, our results showed that cuticle present in growing leaves was ineffective in protecting them from photodamage.

### Mature Leaves Are Able to Cope with Higher Light Arguably Because of a Better Capacity to Export Sucrose and Use Alternative Pathways for Electron Bypass

Considering the parameters derived from the induction of maximum fluorescence, the behavior of F3 mature leaves was in sharp contrast with that of juvenile and growing leaves. Higher illumination did not damage the photosynthetic machinery of mature leaves and did not even trigger adaptive mechanisms such as heat dissipation. We put forward the hypothesis that there was no imbalance between the quantity of energy entering the mature leaves in the form of photon flux and the quantity of energy in the form of electron flux used by the Calvin cycle because the latter was not down-regulated by sugar accumulation (Azcon-Bieto et al., 1983; Stitt et al., 1990), unlike what happened in juvenile and growing leaves. In other words, mature leaves were able to maintain a greater ability to process NADPH, which prevented overheating of the photosynthetic machinery. This fact was displayed in mature leaves by the increase in the dark respiration rate from the 1st day of higher illumination and the following decrease in sugar content.

Numerous studies have shown the negative feedback effect of carbohydrate accumulation on leaf photosynthesis (Azcon-Bieto, 1983; Paul and Foyer, 2001; Paul and Pellny, 2003; Urban et al., 2004; Urban and Alphonsout, 2007). Increased photoinhibition was found to be associated with low sink demand (Buwalda and Noga, 1994; Myers et al., 1999) and the existence of carbohydrate accumulation (Roden and Ball, 1996). Starch accumulation is a mechanism which prevents down-regulation of photosynthesis by providing an outlet for photosynthetic end-products when exports fail to evacuate them effectively. Consequently, starch accumulation can be considered as an indicator of sugar-export efficiency. In mature leaves, the continuous increase in starch content during the treatment, together with the ability to reduce the sucrose content to the level observed before light treatment, indicated excellent sugar-export efficiency. All these data indicated that mature leaves sustained the increased photosynthesis rate induced by higher illumination via starch synthesis and via sucrose export to other plant parts. In juvenile and growing leaves, starch accumulation seemed to cease after 7 and 4 days of higher illumination, respectively, and a high sucrose content was observed during the treatment. Moreover, hexoses accumulated substantially in these leaves, so that the hexose to sucrose ratio was more than twice as high after 9 days of higher illumination. In juvenile and growing leaves, increasing illumination seemed to inhibit sucrose biosynthesis and the subsequent hexose accumulation then reduced the photosynthetic capacity in the leaves.

### In Juvenile and Growing Leaves, the Switch to Higher Illumination Affects PSII Centers Because of Weak NADPH Availability

In juvenile and mature leaves, a net decrease in photosynthesis capacity was found during increased illumination by evaluating the PI and *F*_v_/*F*_M_. As a multi-parametric variable integrating RC/ABS, *F*_v_/*F*_0_ and (1-*V*_J_)/*V*_J_, PI is a much more sensitive and discriminating stress indicator than *F*_v_/*F*_m_ (Živčák et al., 2008). Its decrease is much more pronounced than the decrease in *F*_v_/*F*_M_. In PI, RC/ABS represents the contribution of the density of active reaction (in the sense of quinine acceptor (Q_A_) reducing) centers (on a chlorophyll basis), *F*_v_/*F*_0_ represents the contribution to PI of light reactions for primary photochemistry, i.e., the performance due to trapping probability, and (1-*V*_J_)/*V*_J_ represents the contribution of dark reactions to PI, i.e., the performance due to the conversion of excitation energy into photosynthetic electron transport. Clearly, the lower PI values found in juvenile and growing leaves were attributable to a decrease in RC/ABS and *F*_v_/*F*_0_. The decrease in RC/ABS in these leaves reflected the down-regulation of PSII reaction centers, a well-known mechanism of light adaptation in leaves (Lu et al., 2001). The 33.59 and 23.13% decrease in *F*_v_/*F*_0_, respectively, in F1 and F2 leaves exposed to more intense light for 9 days, suggests that energy trapping probability, in the sense of the quantum yield of primary PSII photochemistry, was indeed reduced. The decrease in (1-*V*_J_)/*V*_J_ values observed in juvenile leaves exposed to increased light may be interpreted as the consequence of a reduced ability to process NADPH, which would impair electron transport capacity at the PSII acceptor side. The PI reduction was particularly noticeable in juvenile leaves, for which the values of the three parameters were statistically different before (day 0) and 7 days after increasing illumination. In many plants, such as sugar beet (Fondy and Geiger, 1982) or *Arabidopsis* (Trethewey and Rees, 1994), dark respiration just after the photoperiod reaches around 50% higher than that of the end of the dark period, due to a greater abundance of hexose and NADPH. The weak increase in dark respiration rate observed in juvenile leaves (or lack of increase in growing leaves) may indicate a higher NADPH consumption rate in these growing organs, indicating weaker NADPH availability attributable to more intense growth.

The adaptive mechanisms triggered by exposure to increased light (down-regulation of energy trapping and conversion, rerouting of electron fluxes and dissipation of excess energy in the form of heat) prove to be insufficient in preventing the photosynthetic machinery from being damaged in juvenile and growing leaves. From 24 h of high light exposure, the initial value of fluorescence *F*_0_ was gradually higher. *F*_0_ is the level of fluorescence emission when all the primary quinone acceptors Q_A_ are in the oxidized state. An increase in *F*_0_ is believed to be caused by the release of free chlorophyll from protein-pigment complexes, which results in blocked energy transfer to the PSII traps (Armond et al., 1980; Sundby et al., 1986; Urban and Alphonsout, 2007). An increase in *F*_0_ may not be reflected in a decrease in *F*_V_/*F*_M_ when there is a concomitant decrease in *F*_M_, which was not the case. Our observations regarding *F*_0_ suggest that the stress associated with the light increase led to photodamage, which is consistent with the previously described effects of severe stress in other plants. For instance, drought stress was observed to induce a loss of the D1 and D2 proteins that constitute the heterodimer core of PSII (He et al., 1995). Taken together, our observations strongly suggest that F2 leaves, and even more so F1 leaves, were not able to cope with higher light, as F3 leaves did, and that some down-regulation and even damage occurred to PSII centers.

### Increased Illumination Affects Growing Leaves due to Inefficient Carbon Metabolism and Weak Photoprotection

Unlike mature leaves, juvenile and growing leaves clearly appeared to have been stressed, as indicated by the PI, to the point of suffering photodamage. Surprisingly, despite tissue organization comparable to that of mature leaves, growing leaves demonstrated high sensitivity to slight light increase. Moreover, the biochemical analysis focusing on phenolics was unable to show differences in HCE and mangiferin content between growing and mature leaves throughout the period of higher light application. Only a late modification in flavonoid content – a severe decrease in (+)-catechin content and a slight increase in rutin content 4 and 7 days after the beginning of increasing light, respectively – contributed to the major differences between growing and mature leaves concerning phenolics. The most significant differences were noted in the carbon metabolism. Growing leaves showed the greatest accumulations of glucose, fructose and starch during the first 7 days of light increase. With a hexose/sucrose rate between 1.5- and 2.5-fold higher than in mature leaves during stress, growing leaves demonstrated an incapacity to convert hexoses, which were found at concentrations that could be toxic for cells (Couée et al., 2006). As end products of photosynthesis, sugars may play a central role in modulating the expression of photosynthesis genes and then in the light acclimation process in growing coffee leaves (Hausler et al., 2014), even though, in mature leaves, inhibition of photosynthesis cannot be attributable to feedback inhibition by carbohydrates (Batista et al., 2012).

### Rutin Accumulation Induced by Increased Illumination in Juvenile Leaf Epidermis Is Too Localized to Offer Effective Photoprotection

Compared to older leaves, glucose and fructose greatly and regularly accumulated in juvenile leaves throughout the treatment and juvenile leaves seemed to be less able to export sucrose. The response of juvenile leaves to higher light was also characterized by a decrease in catechin isomers and mangiferin content and a drastically increased amount of rutin, a UV-absorbing flavonoid. As in growing leaves, a decrease in HCE content was also observed, primarily in adaxial epidermis cells and in spongy parenchyma cells, as indicated by microscopic observations, revealing a loss in leaf antioxidant protection. Rutin synthesis was only found in juvenile leaves and was concentrated in some adaxial epidermis cells and in subsidiary cells of some stomata. In cotton leaves, the accumulation of UV-absorbing flavonoids in epidermal cells has been observed in response to biotic stress (Edwards et al., 2008). Flavonoids, which are produced during the hypersensitive resistant response to *Xanthomonas campestris*, were localized in epidermal cells surrounding bacterial infection sites and protected the living mesophyll cells that surrounded the dead cell cluster from the sunlight-dependent toxicity of the phytoalexins synthesized during the plant response. As in cotton, rutin in juvenile coffee leaves may play the role of a UV screen, but as a chloroplast protectant, more specifically when localized inside the chloroplast. Both *in vitro* and *in vivo* studies have shown that the presence of flavonol glycosides in chloroplasts delays thylakoid lipid peroxidation and correlates negatively with the singlet oxygen level (Takahama and Oniki, 1997; Vanderauwera et al., 2005). Juvenile leaves seemed to respond to high-light stress by a remobilization of phenylpropanoid metabolites for the synthesis of the most active flavonoids to protect the photosynthetic system. These compounds have been considered as a sink for reduced carbon and they may constitute, better than other phenolics, an energy escape valve by consuming triose phosphate, ATP and NADPH (Grace and Logan, 2000; Hernandez and Van Breusegem, 2010). However, unlike what can be observed in *Erigeron breviscapus*, this protection is not efficient in young coffee leaves (Zhou et al., 2016).

## Conclusion

This study showed the limited ability of juvenile leaves to adapt from low to higher light intensity. The present findings obtained in growth chamber conditions are original because, until now, very few works have evidenced contrasted behaviors of leaves according to their developmental stage. They indicate that the lack of acclimation ability of juvenile leaves against stronger lighting could be attributed to ineffective protection. Despite a different response of mature leaves that reflects the acquisition of adaptability during leaf development, the inability of juvenile coffee leaves to develop a useful defense against increased light after initial acclimation to low light clearly emphasizes the understorey origin of the coffee plants. Since most coffee plants are grown under full sunlight, current studies are developed in field conditions to deeply understand ecophysiological and molecular mechanisms involved in coffee tree acclimation to shade culture.

## Author Contributions

CC, HE, LM, and LU participated in the design of the study. J-CB and LT helped CC performing plant cultivation, measurements and sample harvest in growth chamber. DF and SR realized the measurements related to photosynthesis and interpreted the photosynthesis results with LU, CC, and LB. YL and JA performed the evaluation of antioxidant activity and participated to the data analysis. SD, with the help of CL, performed the sugar content analysis. CC and CL, helped by SD, performed starch and secondary metabolite extraction and quantification, LM and PLF performed microscopic analysis, and CC, HE, LB, and BB contributed to their interpretation. CC, LU, and HE wrote the manuscript. All authors read and approved the final manuscript.

## Conflict of Interest Statement

The authors declare that the research was conducted in the absence of any commercial or financial relationships that could be construed as a potential conflict of interest.

## Abbreviations

5-CQA: 5-caffeoylquinic acid, chlorogenic acid
CQA: caffeoylquinic acid
DiCQA: dicaffeoylquinic acid
FQA: feruloylquinic acid
HCE: hydroxycinnamoyl ester
TS: transverse section
