# Supplementary material for: Juvenile Coffee Leaves Acclimated to Low Light Are Unable to Cope with a Moderate Light Increase
Source: Front Plant Sci. 2017 Jul 14;8:1126. doi: 10.3389/fpls.2017.01126 (PMC5509796; doi:10.3389/fpls.2017.01126)

SUPPLEMENTARY MATERIAL

Supplementary Figure S1. Plant material morphology. Aspect and size of the three different leaves studied: juvenile leaf (F1), growing leaf (F2) and mature leaf (F3) of *Coffea arabica* var. Naryelis.

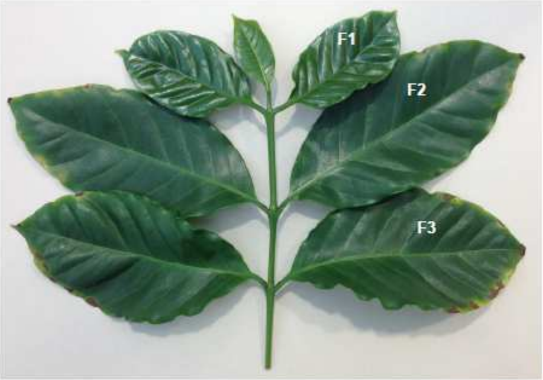

Supplementary Figure S2. Glucose (A) and fructose (B) content evolution in juvenile (F1), growing (F2) and mature (F3) *C. arabica* leaves submitted to an illumination increase (PAR 300 to 500) during 9 days. Error bars represent ± standard deviation of mean (n= 5).

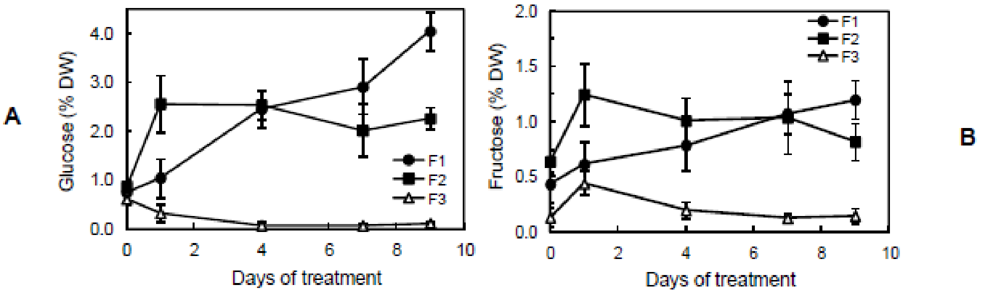

Supplementary Figure S3: Effect of light increase on caffeine content in *C. arabica* leaves under different growth stages (F1: juvenile leaves; F2: growing leaves; F3: mature leaves). Values are the mean with SD of contents from 3 leaves taken on 3 different plants and measured 3 times.

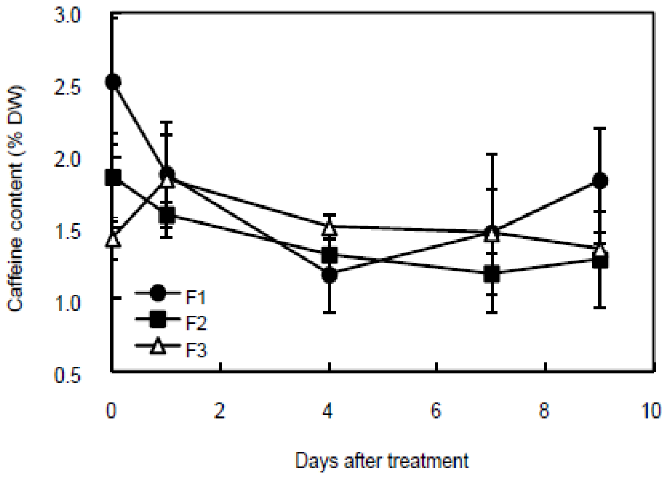

Supplementary Figure S4. Superimposed chromatograms of juvenile (F1) *C. arabica* leaves before (A) and after two days (B) of increased illumination. 1: 3-CQA, 2: 5-CQA, 3: 4-CQA; 4: caffeic acid, 5: FQA, 6: mangiferin, 7:3,4-DiCQA, 8: 3,5-DiCQA, 9: rutin, 10: 4,5-DiCQA.
Gradient separation was carried out on a Agilent Eclipse XDB C18 column with methanol and 2% acetic acid in water as eluents. Detection was done at 360 nm.

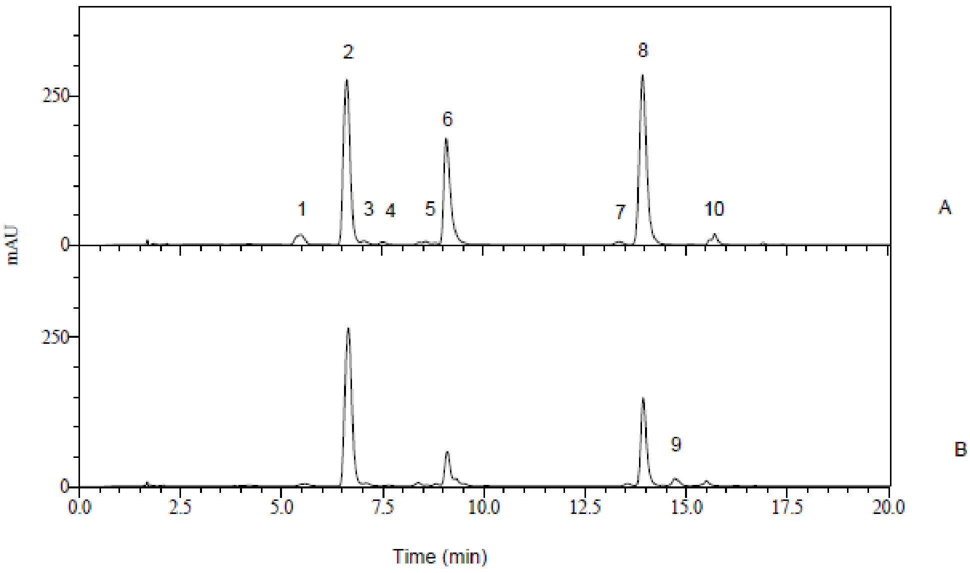

Supplement: Supplementary file 1 [file Data_Sheet_1.PDF]
